# Determination of the Course of Cyan Fluorescence of *Pseudomonas aeruginosa* with a Handheld Bacterial Imaging Device

**DOI:** 10.3390/diagnostics14141474

**Published:** 2024-07-10

**Authors:** Emily Pham, Landrye Reynolds-Reber, Stephany Navarro, Abdul Hamood, Laura M. Jones-Donaldson, Allie Clinton Smith

**Affiliations:** 1 Department of Biological Sciences, Texas Tech University, Lubbock, TX 79409, USA; emily.pham@ttuhsc.edu; 2 Department of Honors Studies, Texas Tech University, Lubbpock, TX 79409, USA; 3 Department of Immunology and Molecular Microbiology, Texas Tech University Health Sciences Center, Lubbock, TX 79430, USA; stephany.navarro@ttuhsc.edu (S.N.); abdul.hamood@ttuhsc.edu (A.H.); 4 MolecuLight Inc., Toronto, ON M5G 1T6, Canada; ljones@moleculight.com

**Keywords:** chronic wounds, *Pseudomonas aeruginosa*, fluorescence, cyan fluorescence, point-of-care, detection, fluorescence imaging

## Abstract

Chronic wound infections are of clinical concern as they often lead to high rates of mortality and morbidity. A point-of-care handheld bacterial fluorescence imaging has been designed to detect the auto-fluorescent characteristics of most clinically relevant species of bacteria. This device causes most species of bacteria to exhibit red fluorescence due to the production of exoproduct porphyrins. One of the most significant contributors to the pathogenicity of chronic wounds is the pathogen *Pseudomonas aeruginosa*, and interestingly, this organism exhibits an additional unique cyan fluorescence signature. There is an over 90% positive predictive value that, when a chronic wound exhibits cyan fluorescence with the bacterial fluorescence imaging device, the wound will harbor *P. aeruginosa*. This project seeks to understand what genetic factor(s) contribute to the cyan phenotype observed.

## 1. Introduction

Chronic wounds are defined as wounds that fail to proceed through the normal stage of wound healing, remaining open for approximately ≤6 weeks [1,2]. While there are many contributing factors that lead to failure in wound healing, one of the main causes of non-healing chronic wounds is the presence of bacterial bioburden, particularly in the form of biofilm [3,4,5,6,7,8]. A biofilm is traditionally defined as the presence of microorganisms, usually polymicrobial, encased in a self-produced matrix [5,6,9,10]. Bacterial presence and proliferation in the chronic wound bed has been demonstrated to contribute to delayed wound healing and other complications [11,12,13] and is often highly polymicrobial [14,15]. The presence of bacterial biofilm and the highly polymicrobial nature of these infections both contribute to the pathogenesis of chronic wound infections.

**Figure 1:**
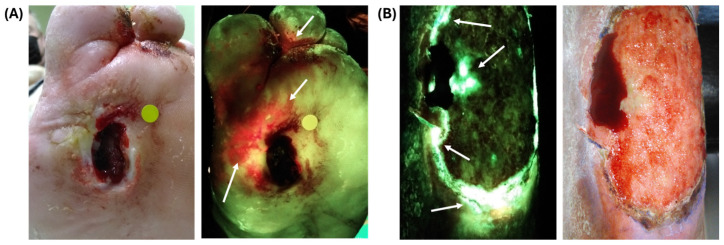
Point-of-care handheld bacterial fluorescence imaging device and detectable fluorescence patterns detectable. (**A**) Diabetic foot ulcer. Standard image (left) and fluorescence image (right) show regions of red fluorescence (as indicated by the white arrow) attributed to heavy growth of porphyrin-producing bacterial species. (**B**) Venous leg ulcer post-debridement. Standard image (left) and fluorescence image (right) show regions with cyan fluorescence, confirmed by culture-based microbiology as heavy burden of *Pseudomonas aeruginosa*, as indicated by white arrows. Images courtesy of MolecuLight, Inc.

A point-of-care handheld bacterial fluorescence imaging device captures images of bacterial wound environments harboring most clinically relevant bacteria by detecting the production of the auto-fluorescent exoproduct, porphyrin [16,17,18] (Figure 1A). This imaging device allows for real-time imaging of wound environments to identify bacterial presence and locate bacterial loads above 10^4^ CFU/g. This allows for improved clinical specimen sampling of chronic wounds [18,19,20,21,22]. Previous studies have demonstrated this porphyrin-dependent red fluorescence in multiple bacterial species, in both in vitro and in vivo experiments [16,23].

One of the most prominent pathogens in the chronic wound environment is *Pseudomonas aeruginosa* [14,24,25]. Jockenhöfer et al. conducted a retrospective analysis of 970 venous legs and detected *P. aeruginosa* in one-third of patients [26], while Gjødsbøl et al. found the prevalence of *P. aeruginosa* to be harbored in more than half of the persisting venous leg ulcers evaluated in their longitudinal study [27]. Chronic wounds harboring *P. aeruginosa* are often larger and experience longer duration than wounds without *P. aeruginosa* [14,25,28,29,30], leading to worse patient outcomes.

In addition to the red fluorescence attributed to porphyrins, *P. aeruginosa* additionally exhibits a unique, cyan fluorescence signature (Figure 1B). Cyan fluorescence induced by violet light in *P. aeruginosa* has been previously noted by our group and others, both in vitro, in vivo, and clinically [31,32,33,34]. *P. aeruginosa* grown on blood agar plates (BAP) exhibit observable cyan fluorescence within 24 h of inoculation [34], and mouse wounds inoculated with *P. aeruginosa* exhibit cyan fluorescence within one day of inoculation, which persists for up to eight days [23].

Clinically, cyan fluorescence is associated with the presence of *P. aeruginosa*, as determined by multiple observational studies and clinical trials using a variety of sampling methods from multiple infection types [35,36,37,38,39,40,41,42]. Hurley et al. examined chronic wounds in an outpatient plastic surgery center and found that all wounds exhibiting cyan fluorescence harbored *P. aeruginosa*, resulting in a sensitivity and specificity of 100% [41]. Raizman et al. conducted a prospective clinical trial examining multiple wound types, and found that 26/28 wounds sampled that exhibited cyan fluorescence were determined to harbor *P. aeruginosa* via culture-based diagnostics, resulting in a positive predictive value of over 90% [34]. Notably, the regions of cyan florescence were detected from regions of low to high growth of *P. aeruginosa*, and less than 20% of these samples that were positive for *P. aeruginosa* via fluorescence imaging exhibited the classic clinical signs and symptoms of *P. aeruginosa* infection [34].

It has previously been hypothesized that this cyan fluorescence signature indicative of *P. aeruginosa* in chronic wounds is due to exoproduct production. Specifically, pyoverdine has been hypothesized to be the source of *P. aeruginosa* cyan fluorescence, due to the previous research surrounding pyoverdine’s intrinsic fluorescent properties [43,44]. However, the source of cyan fluorescence in *P. aeruginosa* has not been definitively determined in previous studies. Pyoverdine production is enhanced under hostile conditions (e.g., low iron) [45,46,47]. For example, an increase in pyoverdine production has been observed in burn wounds [48]. In this study, we seek to definitively determine the relationship between exoproduct production in *P. aeruginosa* (specifically investigating fluorescent pigments, such as pyoverdine) and the observation of cyan fluorescence with the handheld point-of-care imaging device.

## 2. Materials and Methods

### 2.1. Bacterial Strains and Media

Multiple strains of *Pseudomonas aeruginosa* were tested, primarily representing pigment virulent factor exoproducts that are suspected to have fluorescent properties. The first set of mutants are gene knockout mutants originating from the PA14 transposon mutant library [49], using PA14 as the parent strain. These strains are PA14Δ*pchE* (pyochelin synthetase knockout), PA14Δ*phzA* (probable phenazine biosynthesis protein knockout), PA14Δ*phzE* (phenazine biosynthesis protein PhzE knockout), PA14Δ*phzA*Δ2 (probable phenazine biosynthesis protein knockout), and PA14Δ*pvdD* (pyoverdine synthetase D knockout). PAO1 has been previously described [50] and was used in this study as a parent strain. PAO1Δ*pvdS* is a pyoverdine knockout mutant derived from PAO1, exhibiting a 460 base pair deletion in the sigma factor of pyoverdine [51,52]. PAO1Δ*pvdS* lacks this the ability to produce pyoverdine as confirmed by planktonic growth experiments [51,53,54]. These strains were provided to our group by Dr. Abdul Hamood (Texas Tech University Health Sciences Center, Lubbock, TX, USA).

Overnight cultures of bacterial strains were grown in lysogeny broth (LB) (Thermo Fisher Scientific, Dionex, PA, USA) [55] at 37 °C on an incubator shaker at 200 rpm for 24 h. The cells were then taken and standardized to OD_600_ 0.4 nm (~0.5 MacFarland standard) using a spectrophotometer. Standardized cultures were then grown on a modified multimedia (MM9) agar plate [56] and dyed with a 0.25 g/L concentration of black dye (Spectrum 18-602-811, Fisher Scientific) known as bMM9 media. In this study, the agar was dyed black to cut down on autofluorescence from the imaging device.

### 2.2. Fluorescence Imaging

The ~0.5 MacFarland standard concentration of bacteria was quadrant streaked on our in-house bMM9 media. The plates were then imaged using the handheld bacterial imaging device (MolecuLightDX, MolecuLight Inc., Toronto, ON, Canada) at 0 h, 24 h, and 48 h. First, a standard image was taken with the imaging device under ambient lighting. The room lights were then turned off to create the darkened environment required to capture a fluorescence image. The 405 nm excitation light emitting diodes (LEDs) were then turned on, the device was placed 8–10 cm away from the wound (guided by an imaging jig) and a fluorescence image was captured. Specialized optical filters on the device ensure that only relevant fluorescence from tissue and bacteria are captured and the violet excitation light is filtered out. Under violet light illumination, background fluorescence appears various shades of green, while bacteria at loads > 10^4^ CFU/g appear red or cyan [17,18,34,35].

### 2.3. Spectral Emission

For each of the strains grown, the absorbance at OD_600_ nm was collected to standardize spectral data to bacterial growth. From previous inoculation of the strains onto the bMM9 plates and images of the plates using the bacterial fluorescent imaging device, a region was identified on each plate that contained a cyan-positive region and a cyan-negative region, if applicable. An inoculation loop was used to extract the sample from the plates of the cyan-positive and cyan-negative regions and mixed with 100 µL 1X PBS (Thermo Fisher Scientific, PA, USA) in a 96-well spectrofluorometer plate reader, creating a homogenous suspension. The well plate is then excited at 405 nm, and a full emission spectral scan was performed at 435–700 nm in 10 nm increments.

### 2.4. Generation of Complimented Mutant Using Electroporation

Plasmid pVLT31 (broad-host-range expression vector) containing *pvdS* under the tac promoter: Tc^r^ [57].

Plasmid DNA was extracted from PAO/pPVD31 using the Monarch Plasmid Miniprep Kit (New England Biolabs, Ipswich, MA, USA). Plasmid DNA was introduced in to PAO:: *pvdS* by electroporation using a gene pulser (Bio-Rad laboratories, Hercules, CA, USA) to generate PAO:: *pvdS/*pPVD31 [58].

### 2.5. Induction of pPVD31 Using IPTG and Pyoverdine Extraction

For our complementation studies, bacterial strains PAO1, knockout mutant PAO1Δ*pvdS*, and complimented PAO:: *pvdS/*pPVD31 were used. The bacterial strains were grown on lysogeny (LB) broth [55] and trypticase soy broth dialysate (TSBD) [59], which is an iron-limited media; this should stress the bacteria to overproduce virulence factors. PAO1Δ*pvdS* also contained gentamicin (60 µg/mL), and PAO:: *pvdS/*pPVD31 contained gentamicin (60 µg/mL) and tetracycline (80 µg/mL) to maintain the plasmids.

To induce the plasmid of the complimented mutant, the strains were grown in LB or TSBD with the use of IPTG (Thermo Fisher Scientific, PA, USA), a dioxane-free chemical [60]. PAO1, PAO1Δ*pvdS*, and PAO:: *pvdS/*pPVD31 were grown in LB or TSBD for 24 h with the presence of the indicated antibiotics, when required. Overnight cultures were then subcultured twice. After the second subculture, the cultures were diluted 1:100 in 2 mL of LB or TSBD media and subcultured for another 3–4 h at 37 °C on a shaker in the incubator at 220 rpm. After 3–4 h, the cultures were then standardized to OD_600_ nm to receive data for pyoverdine extraction [61]. A total of 1 mL of the remaining cultures were spun down in a microcentrifuge tube at max 14,000 rpm for 30 s to obtain a pellet. The supernatant was removed and saved and measured at OD_405_ nm. This was saved as the uninduced control. The uninduced controls were imaged using the device in standard and fluorescent modes.

The remaining 1 mL of the previous cultures were taken and mixed with the required antibiotic concentrations (when required), 10 µL of 100 mM IPTG, and either LB or TSBD. The IPTG was prewarmed to 37 °C before use. The tubes were then placed in the incubator shaker for 12–16 h at 37 °C. After the incubation period, one 1 mL was taken and measured at OD_600_ nm. The remaining 1 mL from the induced samples were transferred to a microcentrifuge tube and spun at max 14,000 rpm for 30 s to obtain pellets of each strain. The supernatant was then saved and an OD_405_ nm was obtained for the supernatant. This is the induced control. Images of the induced control were then taken by the bacterial fluorescent imaging device in standard and fluorescent modes.

After each of the steps during the induction, a subsample was taken to determine CFU/mL counts at indicated points in the experiment. The 1:100 dilution was plated on LB agar plates with up to 10^−6^ dilution and incubated at 37 °C for 24 h. The 3–4 h dilution was plated on LB agar plates with up to 10^−6^ dilution and incubated at 37 °C for 24 h. The uninduced and induced controls were plated on LB agar plates with up to 10^−6^ dilution and incubated at 37 °C for 24 h.

### 2.6. Statistical Analysis

All experiments were performed in triplicate. Results were averaged with standard deviation shown. Data analysis and calculations were performed in Microsoft Excel (version 2406).

## 3. Results

### 3.1. Investigating P. aeruginosa Knockout Mutants to Determine Which Exoproduct(s) Are Responsible for Cyan Fluorescence

**Figure 2:**
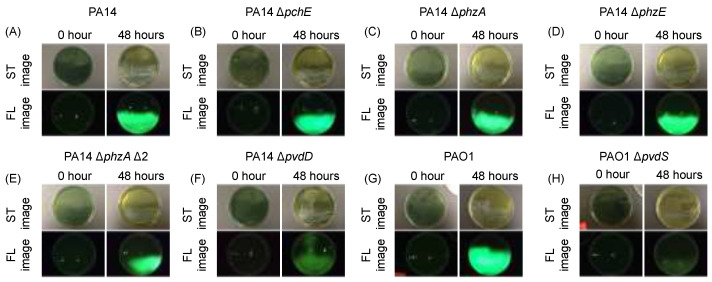
In vitro screening of *Pseudomonas aeruginosa* pigment knockout mutants to observe cyan fluorescent phenotype. *P. aeruginosa* strains were inoculated on modified minimal media 9, dyed black (bMM9), in triplicate. Plates were imaged using the fluorescence imaging device using standard (ST) mode and fluorescent (FL) mode taken at 0 h and 48 h, one representative image shown per condition. PA14 gene mutant bank parent (GMBP) (**A**) and PAO1 (**G**) serves as the parent control for the testing of the knockout pigment genes B–F and H, respectively. Pigment genes tested were knockout mutants of pyochelin (**B**), phenazine A (**C**), phenazine E (**D**), pyocyanin (**E**), pyoverdine synthetase (**F**), and the sigma factor of pyoverdine (**H**).

A series of *P. aeruginosa* knockout mutant strains were generated in order to test their observed cyan fluorescence patterns. The goal of this experiment was to determine which gene(s) need to be disrupted to lose the cyan fluorescence phenotype; this would imply that those gene(s) are required for observed cyan fluorescence with the device. Parent and mutant strains were grown on our in-house bMM9 plates to stress the bacteria to overproduce virulence factors due to the lack of nutrients (specifically iron) in the media The media was dyed black to reduce autofluorescence with the device during imaging. When a series of pigment virulence factor products are tested, observed cyan fluorescence is lost when genes involved in pyoverdine synthesis (PA14Δ*pvdD* and PAO1Δ*pvdS*) were disrupted. These data suggest that pyoverdine expression in *P. aeruginosa* is required for the observed cyan fluorescence phenotype with the imaging device (Figure 2).

### 3.2. Full Spectral Analysis of Knockout Mutants Supports the Hypothesis That Pyoverdine Is Responsible for P. aeruginosa Cyan Fluorescence

Next, regions of observed positive and negative cyan fluorescence from each parent and mutant under investigation were tested to determine the correlation between observed cyan fluorescence with the device to actual cyan fluorescence as determined by full spectral emission. The purpose of this experiment is to validate that cyan fluorescent peaks in the ~435 nm range only exist where observed cyan fluorescence is detected using the device. In essence, we wanted to confirm that that cyan fluorescence (as measured by ~435 nm peaks) were indeed absent in the signal negative regions, not that the cyan fluorescence is not just below a limit of detection with the device.

**Figure 3:**
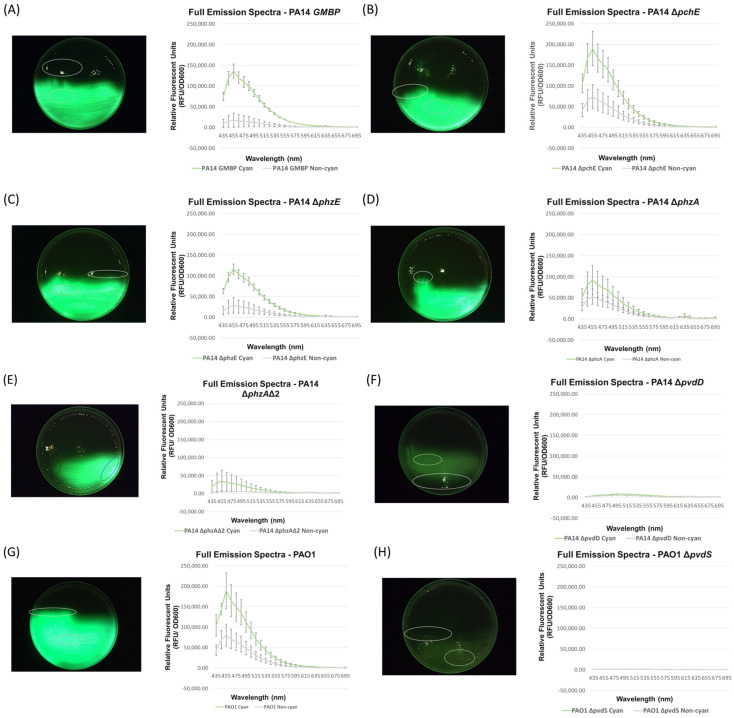
Full Spectral Emission from *P. aeruginosa* Pigment Knockout Mutants. *P. aeruginosa* pigment knockout mutants were screened for cyan fluorescence. Pigment knockout mutants were grown on bMM9 incubated at 37 °C for 24 h in triplicate (one representative image shown), and regions of visible cyan fluorescence and non-cyan fluorescence (where applicable) were harvested (indicated by green and white markers, respectively) and measured for bacterial concentration using OD_600_ nm. Then, full spectral emission was collected using excitation wavelength of 405 nm, emission data captured from 435–695 nm at 10 nm increments. PA14 (**A**) and PAO1 (**G**) were parent positive controls of Δ*pchE*, Δ*phzE*, Δ*phzA*, Δ*phzA*Δ2, Δ*pvdD* (**B**–**F**) and Δ*pvdS* (**H**), respectively.

Our results confirm that where cyan fluorescence is not detected on the surface of the agar plate by the fluorescence imaging device, no cyan peak in the ~435 nm range exists. This indicates that the lack of observable cyan fluorescence with the device is indeed due to a lack of the presence of cyan fluorescence and not due to a limit of detection issue with the device. It is notable that the pyoverdine mutants (PA14Δ*pvdD* and PAO1Δ*pvdS*) exhibit no cyan fluorescence either observable with the device nor on full spectral emission investigation (Figure 3).

### 3.3. Complimenting the Pyoverdine Gene in the P. aeruginosa Knockout Mutant via Plasmid Restores Cyan Fluorescence

Next, a pyoverdine knockout mutant was complemented with a working pyoverdine gene to restore pyoverdine expression. Theoretically, if restoring pyoverdine expression in a knockout mutant then restores the observed cyan phenotype with the imaging device, the data taken together would indicate pyoverdine exoproduct production specifically is responsible for the observed cyan phenotype with the device (and not due to different exoproduct(s)). The PAO1Δ*pvdS* knockout mutant was complimented with pyoverdine gene on plasmid pPVD31 as described in the methods to restore pyoverdine expression in the knockout mutant, named PAO:: *pvdS/*pPVD31. Because the pPVD31 plasmid is under control of the lac operon, induction of expression of the plasmid required an IPTG induction step as described in the methods. This was performed alongside a pyoverdine assay to measure pyoverdine expression, in both complex (LB) and iron limited (TSBD) media.

**Figure 4:**
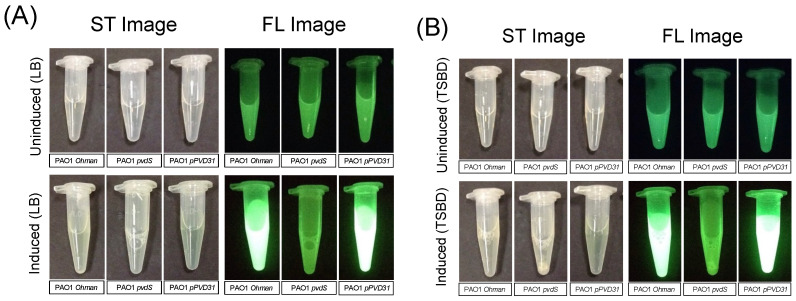
Complementation Experiment with PAO1Δ*pvdS* knockout mutant and complimented mutant PAO:: *pvdS/*pPVD31. Pyoverdine expression was measured using a modified extraction assay as described by Stintzi et al. [61]. PAO1 parent, PAO1Δ*pvdS*, and PAO:: *pvdS/*pPVD31 were incubated in LB (**A**) and iron-deficient TSBD (**B**). The pVLT31 plasmid is under control of the lac operon, so an induction step using IPTG was utilized as described by Hansen et al. [60].

Our results indicate that in both media types, when the parent strain, knockout mutant, and complimented mutant are induced with IPTG and measured for pyoverdine expression, that pyoverdine expression is high, lost, and restored, respectively (Figure 4).

## 4. Discussion

The series of experiments presented demonstrate that when pyoverdine expression is disrupted and lost in wild-type *P. aeruginosa*, cyan fluorescence is no longer detectable with the handheld fluorescent bacterial imaging device. This is not observed when the expressions of other *P. aeruginosa* pigments are altered, suggesting that cyan fluorescence is specific to pyoverdine expression. To confirm the specificity of cyan fluorescence to pyoverdine expression, we complemented the pyoverdine gene back into the PAO1Δ*pvdS* knockout mutant (PAO:: *pvdS/*pPVD31) and demonstrated a restoration of the cyan phenotype.

Pyoverdine expression is specific to a subgroup of Pseudomonads known as the “fluorescent pseudomonads”, comprising *Pseudomonas aeruginosa*, *P. putida*, *P. syringae*, and *P. fluorescens* [62,63]. Therefore, identifying the cyan fluorescence phenotype with the device does not definitively differentiate *P. aeruginosa* from other “fluorescent pseudomonads” in a clinical wound. Nevertheless, considering that *P. aeruginosa* predominates among Pseudomonad species in such wounds [14,24,25,27,64,65], it can be reasonably interpreted that when cyan fluorescence is observed in the wound bed with the device, *P. aeruginosa* is present. This finding is in line with the previous clinical trials calculating the specificity, the sensitivity, and the positive predictive value of the relationship between observed cyan fluorescence and the presence of *P. aeruginosa* in the clinical wound bed [34,41].

*P. aeruginosa* remains a challenging pathogen to treat in chronic wounds. Not only is it the most common and abundant Gram-negative pathogenic bacterium in chronic wounds, but presence of *P. aeruginosa* correlates with larger wounds and a poor prognosis for healing [66]. Thus, rapid detection of this pathogen at point-of-care is critical. These data support the ability to detect this pathogen specifically using the fluorescence imaging device.

For research purposes, having the ability to detect *P. aeruginosa* in real-time without the need for culture-dependent methods (which have a significant delay) or additional diagnostics (like qPCR, which require additional specimen processing and instrumentation) could be beneficial. This device could also be used to understand the environment(s) where *P. aeruginosa* upregulates or downregulates pyoverdine by using the intensity of the observed cyan fluorescence as a real-time proxy for pyoverdine expression.

The results of this study can be used to validate that cyan fluorescence observed in vitro, in vivo, and in the clinical setting is due to pyoverdine expression by *P. aeruginosa*. This finding potentially allows clinicians to diagnose a *P. aeruginosa* infection at the bedside in real-time, independent of time-consuming, culture-dependent methods, which has the potential to improve clinical outcomes.

## Data Availability

Data is contained within the article, further inquiries can be directed to the corresponding author.
